# Impact of diets with a high content of greaves-meal protein or carbohydrates on faecal characteristics, volatile fatty acids and faecal calprotectin concentrations in healthy dogs

**DOI:** 10.1186/1746-6148-9-201

**Published:** 2013-10-09

**Authors:** Ingrid Hang, Romy M Heilmann, Niels Grützner, Jan S Suchodolski, Jörg M Steiner, Faik Atroshi, Satu Sankari, Anu Kettunen, Willem M de Vos, Jürgen Zentek, Thomas Spillmann

**Affiliations:** 1 Department of Equine and Small Animal Medicine, University of Helsinki, Helsinki, Finland; 2 Gastrointestinal Laboratory, Department of Small Animal Clinical Sciences, College of Veterinary Medicine and Biomedical Sciences, Texas A & M University, 4474 TAMU, College Station, TX 77843, USA; 3 Savcor Group Ltd, Mikkeli, Finland; 4 Department of Basic Veterinary Sciences, University of Helsinki, Helsinki, Finland; 5 Laboratory of Microbiology, Wageningen University, Wageningen, The Netherlands; 6 Institute of Animal Nutrition, Section of Veterinary Medicine, Free University Berlin, Berlin, Germany

**Keywords:** Canine, The metabolism of the colonic microbiota, Volatile fatty acids, Faecal canine calprotectin

## Abstract

**Background:**

Research suggests that dietary composition influences gastrointestinal function and bacteria-derived metabolic products in the dog colon. We previously reported that dietary composition impacts upon the faecal microbiota of healthy dogs. This study aims at evaluating the dietary influences on bacteria-derived metabolic products associated with the changes in faecal microbiota that we had previously reported. We fed high-carbohydrate starch based (HCS), [crude protein: 194 g/kg, starch: 438 g/kg], high-protein greaves-meal (HPGM), [crude protein: 609 g/kg, starch: 54 g/kg] and dry commercial (DC), [crude protein: 264 g/kg, starch: 277 g/kg] diets, and studied their effects on the metabolism of the colonic microbiota and faecal calprotectin concentrations in five Beagle dogs, allocated according to the Graeco-Latin square design. Each dietary period lasted for three weeks and was crossed-over with washout periods. Food intake, body weight, and faecal consistency scores, dry matter, pH, ammonia, volatile fatty acids (VFAs), and faecal canine calprotectin concentrations were determined.

**Results:**

Faecal ammonia concentrations decreased with the HCS diet. All dogs fed the HPGM diet developed diarrhoea, which led to differences in faecal consistency scores between the diets. Faecal pH was higher with the HPGM diet. Moreover, decreases in propionic and acetic acids coupled with increases in branched-chain fatty acids and valeric acid caused changes in faecal total VFAs in dogs on the HPGM diet. Faecal canine calprotectin concentration was higher with the HPGM diet and correlated positively with valeric acid concentration.

**Conclusions:**

The HPGM diet led to diarrhoea in all dogs, and there were differences in faecal VFA profiles and faecal canine calprotectin concentrations.

## Background

Bacterial metabolic activity in the large intestine is influenced by the composition of the microbiota, the colonic transit time of the chyme, and the intraluminal pH [1]. It is also closely related to the optimal absorptive functioning of the colon. *In vitro* and *in vivo* studies have shown that the final products of bacterial fermentation mostly depend on the chemical composition of the chyme that reaches the large intestine [2,3]. For instance, if there is a lack of carbohydrates (e.g., wheat, maize) in the diet, the microorganisms ferment amino acids into short-chain fatty acids (SCFAs) and ammonia to obtain energy [4]. A sufficient throughput of carbohydrate leads to a decrease in luminal nitrogenous compounds and an increase in faecal bacterial mass [5]. The chemical composition of the digesta/chyme, the amounts of substrates, and the physical form of the food affect the microbial fermentation [6]. The physical form of the diet depends on the manufacturing process of the diet. Therefore, the latter also affects the characteristics of the microbial fermentation. Evidence for this was provided by a study, on food processing [7]. That study showed that when dogs were fed canned food with poultry and/or beef as the protein source, they had lower total faecal VFAs, but higher levels of faecal valeric, propionic, and branched-chain fatty acids (BCFA) than dogs that were fed commercial dry food with poultry as the protein source [7].

Various studies have shown that diets can impact upon intestinal health and lead to an increased faecal water content and/or high faecal ammonia concentrations. Such changes are mainly due to the protein source [4,8-11] and protein concentration [4,12,13], followed by the carbohydrate concentration of the diet [4,14,15]. The induction of mucosal inflammation is an important pathogenic negative outcome of an impact that diets can have upon intestinal health. Methods for assessing colonic inflammation in humans include the histological evaluation of colonic biopsies and the measurement of faecal biomarkers such as calprotectin, lactoferrin, or polymorphonuclear neutrophil elastase [16,17]. A lack of species-specific faecal biomarkers in dogs has prompted the use of endoscopic sampling of the intestinal mucosa under general anaesthesia for the collection of colonic biopsies [18]. Faecal calprotectin is a Ca^2+^- and Zn^2+^- binding protein that is a sensitive but nonspecific biomarker of intestinal inflammation [19,20]. It is also a protein that has bacteriostatic and fungistatic properties [21,22], which protects the intestinal epithelia against infection and contributes to the innate immunity of the host. Bacteriostatic effects of calprotectin were found against *Listeria monocytogenes*, *Salmonella enterica* spp. *enterica* serovar Typhimurium [23]. Calprotectin is expressed mostly by neutrophils, monocytes [24] and activated macrophages [25], but small amounts were also found to be expressed by keratinocytes [26]. Furthermore, calprotectin levels are increased in serum and other body fluids of patients that suffer from rheumatoid arthritis or from cystic fibrosis, which can be considered to be supporting evidence for its usefulness as a marker of inflammation [27,28] and also for the contribution it makes to the host’s defence mechanism [29]. Recently, a radioimmunoassay for the quantification of canine calprotectin in serum and faeces was developed and analytically validated [30,31].

To the best of our knowledge, experimental studies in dogs that assess canine faecal characteristics, VFAs and their associations with intestinal inflammatory markers are lacking. The current study is the hitherto unreported part of a previously published study [32], in which we investigated the effects of the above mentioned diets on canine faecal microbiota. In that study, we found that faecal samples from the greaves-meal diet fed, dogs favoured growth of species of *Fusobacteriales*. It is important to note that *Fusobacteriales* are potential pathogens for humans [33-35]. Additionally, we reported *Coriobacteriales* in dog faeces: a finding that was reported in only one other recent study [36]. The hypothesis for the current study was that the high protein greaves-meal diet influences the measured faecal parameters, as it was found to remarkably increase the faecal populations belonging to the order *Fusobacteriales*[32]. Therefore, our aim was to study whether the effect of dietary greaves-meal and maize starch on faecal microbiota can be associated with altered faecal characteristics, VFAs, and canine faecal calprotectin.

## Results

The HPGM diet increased the faecal consistency score (*p* < 0.01) and it led to diarrhoea in all the dogs. Faecal pH was higher for the HPGM and the HCS diets than for the DC diet (*p* < 0.01). Faecal ammonia concentrations were (*p* = 0.01) lower for the HCS diet than for either the HPGM or the DC diet feeding periods (Table 1).

**Table 1:** **Analysis of body weight and faecal consistency scores, dry matter, pH and NH**_**3 **_**(n = 3 faecal samples for each variable per diet period) of 5 dogs**

**Variable**	**Baseline (DC)**	**DC**^**1**^	**HPGM **^**2**^	**HCS **^**3**^	**Kruskal-Wallis**	**Mixed model**
					***p*-value**	***p*-value**
**Body weight**, **kg**	19.0 [18.3–19.3] ^A^	19.78 [18.15–21.10]^A^	20.00 [18.60–21.65] ^A^	19.40 [18.80–20.85] ^A^	> 0.10	> 0.10
**Faecal consistency score**	2.5 [2.0–3.0] ^B^	2.5 [2.0–3.0] ^B^	**4**.**0 [4**.**0–5**.**0] **^**A**^	2.5 [2.0–3.0] ^B^	< 0.01	< 0.01
**Faecal dry matter**, **%**	32.2 [30.1–36.2] ^A^	32.4 [30.6–35.4] ^A^	32.2 [13.6–35.9] ^A^	32.5 [29.8–35.7] ^A^	> 0.10	> 0.10
**Faecal pH**	6.9 [6.8–7.1] ^A,B^	6.7 [6.5–7.0] ^B^	**7**.**5 [6**.**6–7**.**9] **^**A**^	7.2 [7.0–7.5] ^A,B^	< 0.01	< 0.01
**Faecal NH**_**3**_**, μg**/**g wet faeces**	1 245 [982–1 482] ^A^	1 079 [693–1 468] ^A,B^	1 191 [710–1 778] ^A^	**835 [657–1**,**162] **^**B**^	0.03	0.01

^**1**^*DC*: dry commercial diet.

^**2**^*HPGM*: high-protein greaves-meal diet.

^**3**^*HCS*: high-carbohydrate starch based diet.

Values are expressed as medians and ranges [min–max]. For each variable, diets not sharing a common superscript (A, B) are significantly different at p < 0.05 and are marked in bold.

The total VFA concentrations were lowest (*p* < 0.01) for the HPGM diet. This reduction could be partly explained by the lower concentrations of acetic acid (*p* < 0.01) and of propionic acid (*p* < 0.01), which both accounted for the largest proportion of the VFAs measured. Although butyric acid was somewhat lower for the HPGM diet compared to the DC diet there was no difference between those diets (*p* = 0.08). Valeric acid and the BCFA concentrations measured were higher for the HPGM diet (*p* < 0.01 and *p* = 0.02, respectively) compared with the DC and HCS diets. The faecal canine calprotectin concentrations remained within the established reference interval [31], but were higher for the HPGM diet (Kruskal-Wallis test, *p* = 0.01) than either the HCS or the DC diet, and they correlated positively with valeric acid (*p* < 0.01).

## Discussion

This study investigated the influence of feeding diets with either high dietary protein (from its greaves-meal content) or high carbohydrate (from its high maize starch content) on food intake, body weight, faecal consistency scores, faecal dry matter, pH, ammonia, VFAs, and levels of faecal canine calprotectin.

None of the three diets used in this study affected food intake or body weight of the dogs differently.

The source and concentration of the dietary carbohydrate and/or protein reaching the colon can alter conditions in the colon, by changing the fermentation characteristics and the faecal microbiota [14,37,38]. Maize and greaves-meal were respectively used in the current study as a source of carbohydrate and protein. It is known from previous studies that maize and greaves-meal are highly digested in the small intestine [8,39,40]. To our knowledge diets with such high maize starch or greaves-meal concentrations have not been used previously in studies.

In our study, the HCS diet did not affect the faecal parameters any differently from the DC diet, except for faecal pH and ammonia. Faecal pH was higher for dogs fed the HPGM diet than when they were fed either the HCS or the DC diets (Table 1). This finding is consistent with those of previous studies, in which protein-rich diets led to increases in faecal pH [7,9]. Faecal ammonia was lowest when the dogs were fed the HCS diet as expected. The ammonia fluctuations could be explained by two mechanisms that require further investigation. First, a sufficient carbohydrate supply to the large intestine in dogs fed the HCS diet, led to a decrease in luminal nitrogenous compounds as reported earlier [5]. Second, when the dogs were fed the HCS diet decreased amounts of protein that reached the hindgut for subsequent fermentation. However, the forenamed mechanisms are hypothetical as the amount of carbohydrates and protein reached to the hindgut was not measured in this study.

There was no dietary effect on faecal dry matter. Nevertheless, the high concentration of dietary protein of the HPGM diet used in our study led to diarrhoea in all the dogs (Table 1). The reason for not reaching statistical significance could be due to the large variation between faecal samples within the same dietary sampling period. This in turn, could be due to the small number of dogs (n = 5) allocated to the study. Further, substantial variations in faecal dry matter between dogs that received the same dietary treatment and a lack of correlation between faecal moisture and faecal consistency score has also been reported in earlier studies [41-43].

Our finding on the undesirable effects of dietary animal proteins on faecal consistency agrees with the results of previous studies [8-12]. Furthermore, the only previous study used bacterial culture methods for the assessment of dietary effects on the faecal microbiota, and it showed an increased number of *Clostridium perfringens* in dogs fed animal (poultry or beef) protein [7]. This is consistent with an increased number of *C*. *perfringens* colonies and decreased counts of *Bifidobacterium* organisms reported in dogs that were fed a low-quality protein diet [8]. In our previous study, the faecal samples obtained from dogs fed the high protein-greaves-meal treatment contained bacterial species that belonged to the orders *Clostridiales*, *Coriobacteriales*, and especially members of the order *Fusobacteriales*[32]. *Fusobacteriales* have been implicated to play a role in many different inflammatory processes, including colonic inflammation [33-35]. Therefore, it could be hypothesised that the diarrhoea observed in all dogs fed the HPGM diet was probably associated with the amount and/or quality of protein, which might change the intestinal microbiota and their metabolic activity [9]. Alternatively, it could be due to the lower levels of carbohydrates that enter the large intestine, which lead to the abundance of bacteria belonging to the order of *Fusobacteriales*[32].

In the present study, the high levels of greaves-meal (the high protein diet) led to a decrease in total VFAs that were mainly attributable to decreases in acetic and propionic acids (Table 2). In contrast, the concentration of faecal valeric acid increased for the HPGM diet. It has been shown in previous studies that there are differences between protein sources in terms of fermentation end products [44], which result in higher proportions of valeric and acetic acids in the canine colon arising from the fermentation of undigested proteins [45]. Acetic acid is produced and excreted by the fermentation activities of acetic acid producing bacteria (e.g., *Acetobacter*, *B*. *subtilis*) [46]. It could be hypothesized that the decrease in faecal acetic acid found in our study for the HPGM diet could be associated with: a) a lower amount of acetic acid bacteria present in the hindgut due to reduced levels of carbohydrate entering the large intestine/colon; or b) it could be due to the high amounts of greaves-meal entering the large intestine. It could also be a combination of both a) and b).

**Table 2:** Canine faecal volatile fatty acid and calprotectin concentrations (n = 3 faecal samples for each variable per diet period) of 5 dogs

**Fecal variable**	**Baseline (DC)**	**DC**^**1**^	**HPGM **^**2**^	**HCS **^**3**^	**Kruskal-Wallis**	**Mixed model**
					***p*-value**	***p*-value**
**Total VFA, mM**	196 [165–221] ^A^	195 [165–239] ^A^	**137 [75**.**0–192] **^**B**^	176 [152.3–198] ^A^	0.01	< 0.01
**Acetic acid, mM**	118 [90.0–135] ^A^	118.9 [93.9–144] ^A^	**71**.**0 [47**.**8–105] **^**B**^	96.9 [83.5–111] ^A,B^	< 0.01	< 0.01
**Propionic acid, mM**	49.3 [45.3–55.9] ^A^	51.1 [33.5–63.8] ^A^	**25**.**2 [7**.**1–31**.**5] **^**B**^	21.1 [14.0–29.9] ^A^	0.02	< 0.01
**Butyric acid, mM**	21.3 [14.8–27.8] ^A^	21.3 [14.8–27.8] ^A^	16.8 [9.0–23.6] ^A^	21.1 [14.0–29.9] ^A^	0.10	0.08
**Valeric acid, mM**	0.4 [0.0–0.5] ^B^	0.4 [0.0–0.8] ^B^	**9**.**8 [0**.**0–15**.**3] **^**A**^	0.6 [0.4–6.6] ^B^	< 0.01	< 0.01
**BCFA, mM**	8.1 [6.8–12.1] ^A,B^	8.3 [5.6–12.4] ^A,B^	**13**.**1 [2**.**2–18**.**9] **^**A**^	8.2 [5.5–12.2] ^B^	0.10	0.02
**Calprotectin**, **μg/g**	4.6 [2.9–7.5] ^B^	3.0 [2.9–20.6] ^B^	**25**.**0 [12**.**0–113**.**4] **^**A**^	8.8 [5.7–22.9]^A,B^	0.01	< 0.01*

*The *ANOVA p*-value was calculated for this variable due to lack of measurement points.

^**1**^*DC*: dry commercial diet.

^**2**^*HPGM*: high-protein greaves-meal diet.

^**3**^*HCS*: high-carbohydrate starch based diet.

Values are expressed as medians and ranges [min–max]. For each variable, diets not sharing a common superscript (A, B) are significantly different at p < 0.05 and are marked in bold.

We also recorded an increase in faecal BCFAs (*p* = 0.02) in dogs fed the HPGM diet. It is generally known that BCFAs are formed by the metabolism of branched-chain amino acids such as valine, leucine, and isoleucine following the breakdown of polypeptides [47,48]. Consequently, the increase in faecal BCFAs measured for the HPGM diet might be simply due to the large amounts of protein entering the hindgut. An increase in the concentration of BCFAs in stools has also been reported in humans who were fed a diet containing supplemental dietary protein based on whey, casein, and lactalbumin [1].

Calprotectin was increased in the faeces of dogs fed the HPGM diet (Table 2) and it was also positively correlated with the concentration of faecal valeric acid. In human medical studies, calprotectin is reported to be a sensitive but nonspecific biomarker of intestinal inflammation [19,20] as the calprotectin parameter is not sensitive enough for the definitive differentiation between underlying intestinal diseases [49-51]. Calprotectin also has bacteriostatic and fungistatic properties [21,22] that have been suggested to protect the intestinal epithelium against infection, thus contributing to the innate immunity of the host. Increased concentrations of faecal calprotectin were detected in patients with Crohn’s disease [20,51], ulcerative colitis [49,50], and colorectal cancer [52]. Recently, the development and analytical validation of a species-specific laboratory method for the measurement of canine calprotectin in faeces for faecal calprotectin has been published [31]. In our study, increases in faecal canine calprotectin concentrations, occurred only when dogs were fed the HPGM diet. This increase also correlated positively with faecal valeric acid levels. Interestingly, the faecal samples from dogs fed the greaves-meal diet had a high preponderance of *Fusobacteriales* bacteria [32]: an order of bacteria that has been suspected to play a pathogenic role in human intestinal inflammation [34-36]. Therefore, one could hypothesize that *Fusobacteria* could produce branched VFAs and valeric acid from protein break-down products [53]. Moreover, some *Fusobacteria* could act as pathogens, which may cause intestinal inflammation that would result in increased faecal calprotectin levels. Therefore, valerate and calprotectin would be expected to be correlated. However, further investigation is needed to assess the possible role of *Fusobacteriales* spp., and increased quantities of bacterially-derived faecal valeric acid and faecal calprotectin in cases of canine intestinal inflammation.

## Conclusions

Feeding a high protein diet with a high content of greaves-meal to dogs led to diarrhoea in all the dogs of our study. Intestinal metabolic (VFA profiles, ammonia, and pH) and inflammatory (faecal calprotectin) parameters for the HPGM diet differed from those of the HCS or the DC diet. We conclude that the HPGM could have mediated the following alternative scenarios: HPGM caused high concentrations of protein to enter the hindgut, HPGM caused lower quantities of maize starch to enter the hindgut, or a combination of both of these. Any of these alternative mechanisms could lead to diarrhoea mediated by changes in the intestinal microbiota and their metabolic activity. Such changes could possibly lead to intestinal mucosal inflammation.

## Methods

### Animals and diets

This study is the hitherto unreported part of a previously published study [32] and was designed as a crossover study. Five adult intact male Beagle dogs^a^ (age: 5 years; body weight: 18–22 kg) were housed individually according to European Union guidelines in indoor-pens (dimension 3.3-6.9 m^2^) at the Experimental Animal Unit of Helsinki University, Finland. The periods included a baseline, the three treatment diets with washout periods. Over the whole study, each dog received all the three diets separated by washout but in different sequence from one another. All dogs entered the baseline period at the same time, followed by a different sequence of dietary treatments and washout periods, which were crossed-over as the study progressed (Table 3). The experimental protocol was approved by the local Ethics Committee for Animal Use and Care in Helsinki, Finland (Animal Experiment Board, Regional State Administrative Agency for Southern Finland, license ESLH-2008-04002/Ym-23). Dogs were exposed to artificial light from 7–16. The environmental temperature indoors was maintained within a range of 15-24°C. The Beagles were considered to be large-framed but not obese as based on visual inspection.

**Table 3:** The feeding sequence of the HPGM, HCS, and DC diets over six feeding periods

**Dog**	**Baseline (DC)**	**Diet (DC**, **HPGM**, **and HCS) and washout periods (WO)**				
**1**	DC diet	DC^**1**^	HPGM^**2**^	WO^**3**^	HCS^**4**^	WO
**2**	DC diet	HPGM	WO	HCS	WO	DC
**3**	DC diet	HC	WO	DC	HPGM	WO
**4**	DC diet	DC	HCS	WO	HPGM	WO
**5**	DC diet	HCS	WO	HPGM	WO	DC

^**1**^*DC*: dry commercial (*DC*) diet.

^**2**^*HPGM*: high-protein greaves-meal diet.

^**3**^*WO*: washout period with the *DC* diet.

^**4**^*HCS*: high-carbohydrate starch based diet.

All dogs received a dry commercial diet^b^ (DC diet comprising: crude protein 264 g/kg; starch, 277 g/kg), a high-protein greaves-meal diet (HPGM diet comprising: crude protein, 609 g/kg; starch, 54 g/kg), or a high-carbohydrate starch based diet (HCS diet comprising: crude protein, 194 g/kg; starch, 438 g/kg) as previously published [32]. In addition to being one of the treatments, the DC was used as the baseline diet and also as the washout diet. Water was provided freely for the duration of the study.

### Sample collection and handling

Fresh naturally-passed faecal samples were collected immediately after defecation on the last three consecutive days of every dietary treatment period for every dog. Each treatment period counted sequentially from d 1 at the beginning of the study baseline as the study progressed to d 129 when the study treatments and sampling ended (Table 3). Thus, the baseline period [Baseline (DC)] on d 10–12, during the diet periods on d 15–17, and for the washout periods on d 22–24. For example, the sampling time points in the feeding sequence for dog 2 (Table 3) were d 10–12 was the end of feeding the baseline DC diet, d 29–31 was the end of feeding the HPGM diet, d 57–59 was the end of first washout period with the DC diet, d 78–80 was the end of HCS diet, d 106–108 was the end of second washout period with the DC diet, and d 127–129 was the end of DC diet period. Samples were thoroughly homogenized and faecal pH was measured immediately after collection. Several 1-g aliquots were then placed in preweighed sterile Sarstedt faecal collection tubes^c^ and frozen at −20°C for further analyses of ammonia, dry matter, and VFAs. A subsample was frozen at −80°C for the measurement of faecal canine calprotectin.

For ammonia measurement, 1-g faecal samples were pretreated by thorough mixing with 3 mL of 1 mol/L HClO_4_ before freezing at −20°C. Faecal samples frozen at −80°C were shipped on dry ice to the Gastrointestinal Laboratory at Texas A&M University, USA for further analyses of faecal canine calprotectin concentrations.

### Clinical parameters

Body weight, food intake, and faecal consistency scores were determined daily, as described previously [54]. Dogs were weighed once a week in the morning, before feeding. For food intake assessment, the amount of food allocated to each dog was calculated according to the individual energy requirements for each dog before entering the study. The amount of food given in grams was weighed before it was given to each dog. Food weighing after feeding was not needed as all the dogs ate everything at every feeding point.

### Fecal parameters

Faecal dry matter, pH, ammonia, VFA profiles, and canine calprotectin concentrations were measured in all faecal samples. Faecal dry matter was determined by overnight oven-drying of each sample at 103°C, as previously described [7]. Faecal pH was measured by using a digital pH-meter^d^, as previously described [7,55]. In order to ensure accuracy, the means of 3 replicate measurements were calculated and entered into the data analysis. Faecal ammonia concentrations were measured by an enzymatic method [56], using an ammonia assay kit^e^ adapted for an automated chemistry analyzer^f^, according to the manufacturer’s instructions.

The faecal VFA profiles, had determinations of propionic acid, acetic acid, butyric acid, valeric acid, and BCFA (isobutyric and isovaleric acid) and were measured, using a modified version of previously described protocols [7,57] as follows: 0.5 g of faeces were mixed with 4.25 mL of deionized water and 250 μL of a premade solution of an internal standard^g^, the mixture was then vortexed for 4 min, centrifuged at 5000 × *g* at +4°C for 15 min, followed by centrifugation of 1 mL of the supernatant at 10 000 × *g* at +4°C for 10 min. The clear supernatant was then filtered into a 1.5-mL crimp vial by using a syringe filter^h^. For measuring VFA concentrations, 1 μL of the filtrate was used for gas chromatography with a flame ionization detector^i^.

Faecal calprotectin concentrations were determined at the Gastrointestinal Laboratory at Texas A&M University. The faecal samples for all dogs (n = 5) for calprotectin determinations were collected on the last three consecutive days at the end of each dietary period then pooled and thoroughly mixed. A previously developed and analytically validated in-house radioimmunoassay was used [31].

### Data handling and statistical analyses

Data are presented as medians and ranges for the minimum and maximum and were analyzed by Excel and JMP 7.0. The data of samples taken for the last three consecutive days at the end of each diet period were assessed for all parameters analysed, except faecal calprotectin concentration. For each dog, the means of food intake, body weight, and faecal consistency score, faecal dry matter, pH, ammonia, and VFAs for three days were calculated and each parameter entered as a single datum. The faecal calprotectin concentrations represent one pooled sample of three consecutive days (one faecal sample per day) at the end of each diet period. The significance of the dietary effect was determined, using a mixed-effect model that included the parameters dog, dog X diet interaction, and the time nested under the diet as random effects. The non-parametric Kruskal-Wallis test was used for evaluating the differences among the diet treatments. Pair-wise Spearman correlation coefficients were calculated for all parameters measured. Unless otherwise stated, the *p*-values reported refer to the mixed-effect model. Significance was set at below 0.05 for all statistical analyses.

## Endnotes

^a^Beagle dogs, origin: Harlan-Winkelmann, Borchen, Germany;

^b^Mastery Pro Adult Maintenance, Raili Pispa Oy, Muurla, Finland;

^c^Faecal collection tube (101 × 16.5 mm; incl. spatula), Sarstedt Oy, Vantaa, Finland;

^d^Knick pH-Meter 761 Calimatic, Knick Elektronische Messgeräte GmbH & Co, KG, Berlin, Germany;

^e^Ammonia Assay Kit, Megazyme International Ireland Ltd., Wicklow, Ireland;

^f^KONE Pro, Thermo Fisher Scientific, Vantaa, Finland;

^g^Internal standard: 140 mM 4-methylvaleric acid in formic acid, Helsinki, Finland;

^h^Acrodisc LC 13 mm with a 0.2-μm polyvinylidene fluoride (PVDF) membrane, 4450 T, Pall Corporation, Port Washington, New York, USA;

^i^Agilent 7890A and 7683, Agilent Technologies, Espoo, Finland.

## Abbreviations

HClO4: Perchloric acid.

## Competing interests

The authors declare that they have no competing interests.

## Authors’ contributions

IH, WMV, JZ and TS conceived the study design; IH performed the sample collection; NG, RMH, JSS and JS performed the faecal calprotectin analysis; IH and SS performed all analyses except for the faecal calprotectin analysis; AK and IH performed the statistical analyses; all of the authors contributed to the writing of the manuscript. All authors read and approved the final manuscript.
